# Interference of Implicit Causality in Relative Clause Processing

**DOI:** 10.1162/opmi_a_00193

**Published:** 2025-03-03

**Authors:** Céline Pozniak, Barbara Hemforth

**Affiliations:** Structures Formelles du Langage, University of Paris 8, CNRS, Paris, France; Laboratoire de Linguistique Formelle, City University of Paris, CNRS, Paris, France

**Keywords:** implicit causality, relative clauses, French, acceptability judgments, self-paced reading

## Abstract

Differences in the processing of subject and object relative clauses have been explained by a combination of syntactic, semantic, and pragmatic factors, such as a general subject advantage based on syntactic constraints, effects of animacy, and the discourse status of relative clause internal subjects. In this paper, we will focus on a factor related to verb meaning, the implicit causality of the verb, which biases the principal causer of the event described by the verb. Depending on whether the bias is on the subject or the object, implicit causality can conflict with the foregrounded antecedent of the relative clause, leading to increased difficulty in comprehension. We tested this hypothesis by manipulating implicit causality in subject and object relative clauses. We used both offline (acceptability judgment task) and online (self-paced reading task) methods to observe at which stage of processing implicit causality influences comprehension. Our findings from acceptability judgments showed that object relative clauses with subject-biased verbs were the least acceptable and the least understood. Conversely, object relative clauses with object-biased verbs were as acceptable and easy to understand as subject relative clauses in French. However, results from self-paced reading indicated that subject-biased verbs were more difficult to process regardless of the construction, suggesting that the integration of implicit causality occurs at a later level of processing, such as in acceptability judgments and comprehension questions. Further acceptability judgment tasks suggested that implicit causality influences relative clause acceptability beyond word order and thematic roles. We propose linking the role of implicit causality with the function of a restrictive relative clause and introduce the Aboutness Hypothesis to explain relative clause processing: a relative clause is more acceptable and easier to understand when everything contributes to making the head its optimal aboutness topic.

## INTRODUCTION

Relative clause processing is a highly studied phenomenon in the psycholinguistic literature. This paper contributes to this literature with evidence from relative clause processing in French. On more theoretical grounds, our experiments address two major questions: First, we ask the more basic question of whether and at what stage in processing so-called implicit causality biases of verbs influence the comprehension of relative clauses. Second, in the general discussion of our data, we want to suggest an explanation of why we find these effects, proposing a hypothesis based on the status of the antecedent as the aboutness topic of the relative clause.

When we interact in communication, we typically try to provide the information necessary for the listener to arrive at the best possible understanding of the message we want to convey (Grice, 1975). As rational speakers (Frank & Goodman, 2012), we choose the constructions that optimize this transfer of information. From this perspective, each construction has a particular function in the current discourse, and this function needs to be taken into account to estimate what makes it more or less easy to process. In this paper, we will focus on early and late stages of processing of restrictive relative clauses, and, in particular, in how far their function influences ease of processing through the role of the implicit causality of the verb. Restrictive relative clauses usually modify a nominal antecedent (Abeillé & Godard, 2021; Bianchi, 2002)^1^ and convey information about it that can be used to identify the corresponding referent in the current discourse universe (Fox & Thompson, 1990). For example, in (1), the relative clause provides information about the *lawyer* and helps the reader/listener to identify which lawyer the sentence is about. The antecedent can have the function of the subject within the relative clause as in (2) for subject relative clauses or the object as in (3) for object relative clauses^2^. This implies that what the relative clause is about will be its antecedent, here the lawyer in both (2) and (3).

(1)  L’avocat qu’elle connait va  au  restaurant.
    The lawyer that she knows is going to the restaurant.
    ‘The lawyer that she knows is going to the restaurant.’
(2)  Subject Relative
    L’avocat [_CP_ qui connait le professeur] va  au  restaurant.
    The lawyer [_CP_ that knows the teacher] is going to the restaurant.
    ‘The lawyer that knows the teacher is going to the restaurant.’
(3)  Object Relative
    L’avocat [_CP_ que le professeur connait] va  au  restaurant.
    The lawyer [_CP_ that the teacher  knows] is going to the restaurant.
    ‘The lawyer that the teacher knows is going to the restaurant.’

As such, a topic regularly debated in the psycholinguistic literature is the difficulty in relative clause processing depending on the function of the antecedent in the relative clause. Keenan and Comrie (1977)’s Accessibility Hierarchy suggests that subject relative clauses are typologically more accessible than object relative clauses in many languages. In line with these observations, previous research has shown that subject relative clauses as in (2) are generally easier to process than object relative clauses (3) in many languages, like Dutch, French, German, English (e.g., Mak et al., 2002, 2006 for Dutch; King & Just, 1991 for English; Schriefers et al., 1995 for German or Frauenfelder et al., 1980; Holmes & O’Regan, 1981 for French a.o.; see Hsiao & Gibson, 2003 for conflicting evidence in Mandarin Chinese and for a review of the literature on relative clause processing, see Lau & Tanaka, 2021).

The core dispute over the years has been about which factor or combination of factors can explain this difference in processing. The factors considered in the literature can be classified into three main categories: syntax-based factors, memory-based factors and semantic/discourse-based factors. The psycholinguistic literature shows that all these factors play a role in the processing of relative clauses at some stage, interacting with each other, thus raising questions about the universal subject advantage and rather favoring the idea of multiple constraints leading to considerable variation with respect to the ease of relative clause processing within and across languages (Gennari & MacDonald, 2008; Kidd et al., 2007).

In our paper, we propose another constraint that may play a role by focusing on the implicit causality of the verb. We hypothesize that the implicit causality of the verb influences the processing of relative clauses in the following way: We predict that object relative clauses with subject-biased verbs will be more difficult to accept and to understand than those with object-biased verbs. We will suggest that this happens because of a conflict of foregrounding, i.e., what the relative clause is about.

In this introduction, we will first review three categories of factors for relative clause processing difficulty. Then, we will present how implicit causality may modulate relative clause processing difficulty.

### Previous Theories on Subject and Object Relative Clause Processing

Some (but not all) syntactic theories have argued for a so-called universal subject advantage, which they explain in different ways.

Accounts based on structural distance as stated in the Relativized Minimality hypothesis (Adani et al., 2010; Friedmann et al., 2009; Rizzi, 1990) claim that the longer the structural distance between the filler and the gap in the syntactic representation of the sentence, the more difficult the relative clause is to process. Since the gap in the object relative clause is always structurally more embedded than in the subject relative clause, object relative clauses should be more difficult to process (see (4) and (5))^3^. Moreover, the subject intervening between the gap and the filler in the object relative clause would add even more processing complexity depending on the amount of grammatical features shared with the modified object.

(4)  Simplified structural representation of subject relative
    
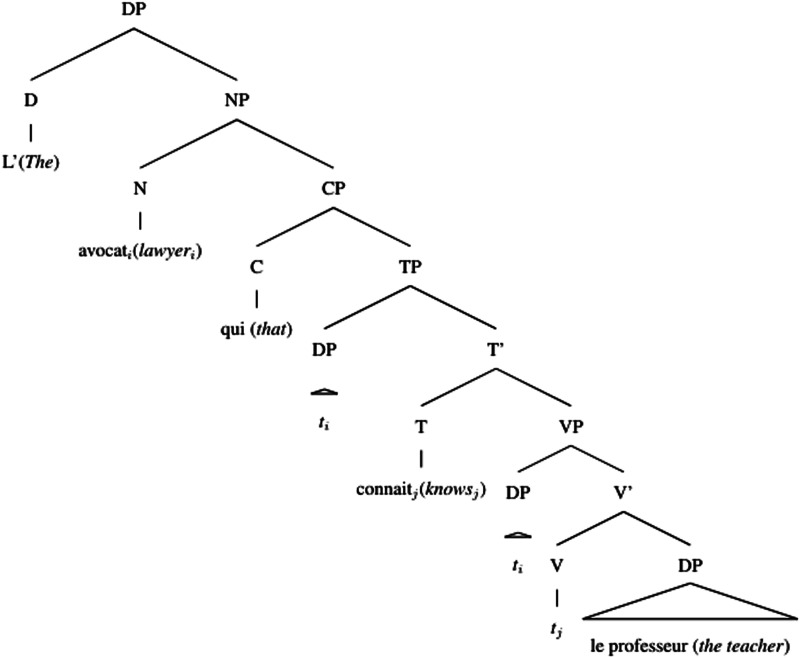
(5)  Simplified structural representation of object relative
    
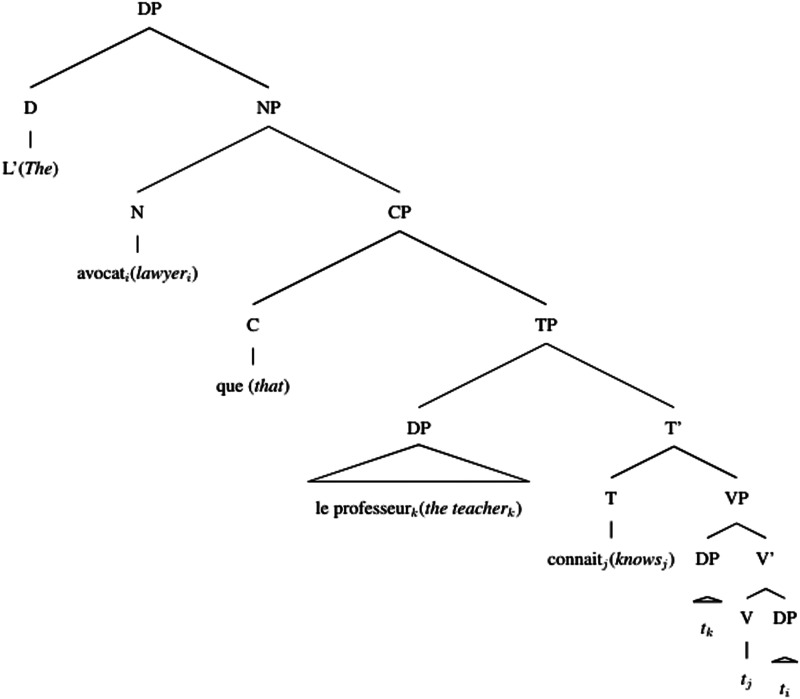

While the theories discussed so far predict the subject advantage to be universal based on syntactic factors, other theories predict that the asymmetry between subject and object relative clauses results from processing issues based on memory-based factors.

The similarity-based interference theory (Gordon et al., 2006) predicts difficulty in processing when, while reading a sentence, we need to hold several similar noun phrases (NPs) in working memory before integrating one of them with a verb (see also Lewis et al., 2006 for similar predictions). Object relative clauses such as (3) should therefore be more difficult to process than subject relative clauses since two similar noun phrases have to be held in memory before one of them can be semantically and syntactically integrated with the verb. Another memory-based theory, the Dependency Locality Theory (Gibson, 1998, 2001) predicts difficulty in processing relative clauses focusing on the linear distance between the antecedent of the relative and the gap rather than the similarity between the noun phrases ((6) and (7)). Predictions of memory-based theories are, however, mostly equivalent across many languages: an advantage for subject relative clauses in processing for languages with postnominal relative clauses and SVO order like English, Dutch, German and French (for relative clauses with preverbal subjects)^4^.

(6)  Linear representation of subject relative
    
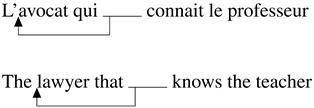
(7)  Linear representation of object relative
    
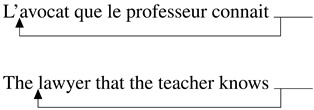

The universality of the subject advantage has also been challenged by a variety of theories that take into account semantic and discourse constraints to predict differences within languages in relative clause processing.

An important constraint would be animacy: object relative clauses appear to be less difficult to process depending on the animacy of the antecedent. Previous research has actually found that they can be very easy to process when there is a difference in animacy between the noun phrases. In particular, object relative clauses were found to be easier when the antecedent is inanimate and the subject in the relative clause is animate (8) compared to when the antecedent is animate and the relative clause subject is inanimate (9) (Frauenfelder et al., 1980 for French; Mak et al., 2002 for Dutch; Gennari & MacDonald, 2008; Traxler et al., 2005 for English).

(8)  The movie that the director watched received a prize.
(9)  The director that the movie pleased received a prize
            (Gennari & MacDonald, 2009, p. 3)

Moreover, thematic roles, interacting with animacy, seem to play a role in object relative clause processing. In six studies involving corpus observation, comprehension and production experiments, Gennari and MacDonald (2009) robustly found that object relative clauses with what they call theme-experiencer (mostly stimulus-experiencer psychological verbs like *annoy* or *please*) verbs (10) were more difficult to process than those with agent-theme verbs (11) (agent-patient verbs like *hire* but also agent-evocator verbs like *criticize*, Bott & Solstad, 2014)^5^. Thematic roles are obviously strongly related to the question of implicit causality and we will come back to their role in more detail in our Experiment 4.

(10)  The writer that the critic angered had written novels …
(11)  The writer that the critic analyzed had written novels …
            (Gennari & MacDonald, 2009, p. 14)

According to Mak et al. (2006), having an animate antecedent is not the decisive factor in relative clause processing, but rather the topicwhorthiness of the internal NP.

To understand Mak et al. (2006)’s argumentation, it is important to differentiate aboutness (or sentence) topics from discourse topics. Aboutness topics refer to what the sentence (or the clause) is about. For example, in (1) the sentence (as well as the relative clause) is about *the lawyer*, making it the aboutness topic. These topics are strictly defined at the sentence level and can be syntactically restricted in some languages, such as German (Frey, 2007). Discourse topics, on the other hand, refer to what a connected part of speech or text is about, meaning they will have been mentioned in the previous discourse. This is evident in (1) where the pronoun *she* refers to someone already mentioned in the discourse (background information), likely making it the discourse topic. While discourse topics are discourse-given, aboutness topics can coincide with the discourse topic in cases of topic continuity. However, they can also differ from the discourse topic in situations of topic shift (Bianchi & Frascarelli, 2010). For instance, in (12), the sentence topic initially coincides with the discourse topic (*Peter*), but then shifts to indicate a topic change with *Peter* as the discourse topic and *Mary* as the aboutness topic in the third sentence. Therefore, a sentence indicating a topic shift can have an aboutness topic that differs from the discourse topic.

(12)  Peter went to the restaurant. He ordered the chef’s speciality. Mary didn’t like this option and went for a salad instead.

In the case of a restrictive relative clause, the antecedent is necessarily the aboutness topic of the relative clause (Krifka, 2008; Kuno, 1976), meaning that the relative clause is about the antecedent. Given the function of a restrictive relative clause in identifying an antecedent in the current discourse, its antecedent is rather unlikely to be the current discourse topic which is by definition already highly active and accessible, and mostly part of the background information. However, from this perspective, relative clause internal noun phrases (which are not the aboutness topic) can easily refer to information already mentioned in the context, and thus be discourse topics, making them backgrounded information in the restrictive relative clause. This is exemplified by the pronoun *she* in (1) (Krifka, 2008). Accordingly, the antecedent of a restrictive relative clause must be the aboutness topic of the relative clause.

Mak et al. (2006) suggest that the topic status of the antecedent should be considered in relative clause processing to explain a general subject relative clause advantage. Since the antecedent is the aboutness topic of the relative clause, and, by default, topics tend to be the subject of a clause, other things being equal, the antecedent will be preferred to have the subject function in the relative clause. This would explain a subject relative clause advantage when the antecedent and the relative clause internal noun phrase have the same animacy status. Consequently, they proposed the *topichood hypothesis*: the noun phrase chosen as the subject of the relative clause will depend on its topicworthiness. For the authors, the topic status of the antecedent, animacy and verb semantics all contribute to topicworthiness.

As for object relative clauses, Mak et al. (2006) suggest that, here, two candidates compete for the status of the aboutness topic of the relative clause: the antecedent and the relative clause internal subject, since subjects are generally seen as the default aboutness topic of a sentence. The competition between two potential topics would then explain the disadvantage of object relative clauses. According to Mak et al. (2006), however, having a relative clause internal subject can be justified when it is sufficiently topicworthy. It may not be very clear why increased topicworthiness of the internal subject does not make the competition worse. It is important to note here that in Mak et al. (2006) no distinction is made between discourse topics and aboutness topics. Only topicworthiness of the internal subject as a discourse topic is taken into account. The authors provide empirical evidence that having a discourse-old relative clause internal subject can facilitate object relative clause processing. A relative clause internal subject that is the discourse topic like the pronoun in (13) is more discourse topicworthy than a noun phrase that is not the discourse topic. This justifies its role as the subject of the relative clause, making the relative clause easier to process compared to (14). An alternative explanation could be that a strong clause internal discourse topic enters less into competition with an antecedent that needs to be the aboutness topic.

(13)  The car that she borrowed had a low tire.
            (Mak et al., 2008, p. 172)
(14)  The car that the teacher borrowed had a low tire.

Mak et al. (2008) and Roland et al. (2012) found that object relative clauses were easier to process when the subject in the relative clause has been mentioned before and put in a topic position in the preceding discourse^6^. The difference between subject and object relative clauses will depend on whether the relative clause internal noun phrase is the discourse topic or not (with a relative clause internal discourse topic making processing easier for object relative clauses). This topichood hypothesis is thus considered as subsuming other factors such as animacy, thematic roles and topicality. The proposal we make in this paper is very much in line with this account.

### Linking Relative Clause Processing With Implicit Causality

While numerous factors have been explored to explain relative clause processing, the implicit causality of the verb has been largely overlooked. Implicit causality is a feature of some verbs that bias for the principal causer of the event they describe (Caramazza et al., 1977). In an SVO sentence, the verb foregrounds either the subject or the object as the principal causer, classifying the verb as subject-biased or object-biased. For example, in (15), the verb *trouble* is subject-biased, attributing causality to the subject (the lawyer has done something causing the trouble). In contrast, in (16) the verb *hate* is object-biased, foregrounding the object (the teacher has done something justifying the hate). Typically, a causal continuation of the discourse will relate to the subject for subject-biased verbs (the lawyer did something) and to the object for object-biased verbs (the teacher did something). Implicit causality thus introduces the probable topic of the upcoming discourse. The reasons for these causality biases are still debated. Bott and Solstad (2014) attribute implicit causality biases to an empty slot for an explanation that is part of verb semantics (at least for psychological verbs and so-called agent-evocator verbs), while van den Hoven and Ferstl (2018) argue that speakers’ knowledge of the world and discourse context underlie verb biases. Importantly, the next-mention bias triggered by the verb meaning does not depend on an explicit causality connector although connectors obviously play an important role. Koornneef and Sanders (2013) demonstrate that connectors like the adversative but can reverse the preferences. However, Kehler et al. (2008) finds that explanations are highly expected after implicit causality verbs even without an explicit connector, and next-mention probabilities follow implicit causality biases in these cases. We can thus assume that implicit causality biases of verbs increase the prominence of subjects or objects independent of the presence of a causality connector.

(15)  Example of subject-biased verbs
    
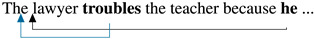
(16)  Example of object-biased verbs
    
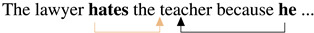

While most studies in the literature focus on explaining the impact of implicit causality on pronoun resolution or next-mention probabilities in the discourse (see Kehler et al., 2008; Koornneef & Van Berkum, 2006, among others), as well as relative clause attachment (see Rohde et al., 2011), to our knowledge, this factor has not been studied in relation to relative clause processing. There is of course some evidence with respect to thematic role effects (Gennari & MacDonald, 2008) that may contribute to this question. Stimulus-experiencer verbs that seem to make object relative clauses particularly hard to process are strongly subject-biased. We argue that this subject bias is the underlying reason for the processing disadvantage.

In this paper, we aim to revisit the debate between subject and object relative clause processing by combining empirical evidence for effects of verb biases with an explanation in line with Mak et al. (2006)’s discourse-based account.

As we stated before, the antecedent is the aboutness topic of a relative clause, i.e. what the relative clause is about. The implicit causality bias of the verb seems to be relevant in this respect by foregrounding the subject or the object of the relative clause increasing its status as a potential topic by making it more salient. Implicit causality bias may thus interfere with the aboutness topicworthiness of the antecedent.

If we take into account the implicit causality of the relative clause verb, we can predict different levels of difficulty in processing depending on the combination of relative clause type and verb bias. Concretely, relative clauses as in (17)–(20) all provide some information about the antecedent, here *the lawyer*, whether it is a subject relative clause or an object relative clause. As for implicit causality, which noun phrase will be foregrounded depends on the verb bias: *the teacher* in (18) and *the lawyer* in (20) in beige for the object-biased verb, and *the lawyer* in (17) and *the teacher* in (19) in blue for the subject-biased verb.

(17) 
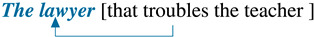
(18) 
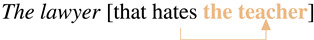
(19) 
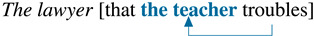
(20) 
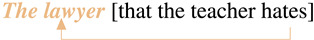

From this perspective, the foregrounding conveyed by verb bias and relative clause type will conflict in object relative clauses with subject-biased verbs and in subject relative clauses with object-biased verbs, thereby increasing processing difficulty. For the object relative clause in (19), the subject-biased verb *trouble* foregrounds the subject *the teacher*. This conflicts with the necessity of having the relative clause antecedent (object) *the lawyer* foregrounded. We predict that this conflict will make comprehension of object relative clauses especially difficult compared to the object relative clause in (20) where verb bias and relative clause type align. In (20), the object-biased verb *hate* foregrounds the object *the lawyer* as does the object relative clause. We expect object relative clauses with subject-biased verbs to be harder to process than object relative clauses with object-biased verbs because of this conflict of foregrounding.

As for subject relative clauses as in (17) and (18), differences in processing difficulty between subject-biased verbs and object-biased verbs may also be expected, but possibly to a lesser extent because of the special status of subjects as default topics. Subjects typically constitute the ongoing topic of the discussion (as stated in the *topichood hypothesis* suggested in Mak et al., 2006, 2008; Roland et al., 2012). The property of being “about” something is also inherently associated with subjects by default (Cook & Bildhauer, 2011; Krifka et al., 2007). Therefore, verb bias may play a less important role in subject relative clause processing.

Verb bias can either reduce or increase the conflict of topicworthiness between the antecedent and the relative clause internal subject. We predict that object relative clauses with subject-biased verbs will increase processing difficulty and reduce acceptability due to the conflict over what the relative clause is “about”. Conversely, object relative clauses with object-biased verbs are predicted to be easier to process since there is no conflict in foregrounding. An empirical question for this paper is that of when implicit causality influences processing, whether in early measures such as reading times or in later measures like acceptability or accuracy in comprehension questions. Earlier work Gennari and MacDonald (2008) may suggest the possibility of early effects.

This paper is organized as follows. In Experiments 1 and 2, we directly tested the effect of implicit causality through offline and online methods. Specifically, we aimed to determine at which stage processing difficulty occurs—if any difficulty arises. Results from acceptability judgments (Experiment 1) showed that verb bias modulated the difficulty of object relative clauses, making them more acceptable with object-biased verbs. Implicit causality did not clearly impact subject relative clauses. Results from a self-paced reading task (Experiment 2) indicated that while reading times on the main verb region (following the relative clause, parallel between conditions) were longer for subject-biased verbs regardless of the relative clause type, the effects of verb bias on accuracy and question answering reaction times became apparent at a later stage of processing. Specifically, accuracy was lower and reaction times for question answering were longer with subject-biased verbs for object relative clauses than for object relative clauses with object-biased verbs and for both verb bias conditions in subject relative clauses. This pattern of results suggests that verb bias interacts with clause type at a later stage of processing.

In the next two experiments (Experiments 3 and 4), we explored the potential influence of factors other than implicit causality on relative clause processing. One such factor is the presence of an intervening subject, which has been frequently invoked to explain the reduced acceptability and processing difficulty of object relative clauses with preverbal subjects. This syntactic explanation could be reinforced by implicit causality and might also explain the lack of an effect observed for subject relative clauses. To investigate this, we conducted an acceptability judgment task on object relative clauses with preverbal subjects and postverbal subjects in French. If the intervening subject plays a major role, we would expect a smaller effect of implicit causality in object relative clauses with postverbal subjects compared to those with preverbal subjects. Specifically, we should not observe strong differences between object relative clauses with postverbal subjects and subject-biased verbs, and object relative clauses with postverbal subjects and object-biased verbs. However, the results from acceptability judgments indicated that verb bias contributed to acceptability difficulties for object relative clauses regardless of the subject position.

Next, in Experiment 4, we discussed another factor: the influence of thematic roles on relative clause acceptability. Previous studies, such as Gennari and MacDonald (2009) have found that the thematic roles of the relative clause verb can modulate the processing of object relative clauses. Since thematic roles and implicit causality often overlap, it can be challenging to determine which of the two factors plays a major role. To address this, we conducted another acceptability judgment experiment to ascertain whether implicit causality can be established as a factor distinct from thematic roles. Our data suggest a strong correlation between biases and thematic roles. Finally, we discuss our results and suggest an hypothesis combining verb biases and discourse constraints to explain them based on the antecedent topicworthiness (the Aboutness hypothesis).

## EXPERIMENT 1

To test predictions regarding the effect of implicit causality on relative clause acceptability and comprehension, we first conducted an acceptability judgment task in French, followed by a self-paced reading task to observe its influence on early processing. It is important to note that French relative clauses do not exhibit any local ambiguity, distinguishing them from their English counterparts. While English subject and object relative clauses are disambiguated only after the relative pronoun (*that*) (Traxler et al., 2002), French relative clauses are unambiguous from the outset, with *qui* marking subject relative clauses and *que* marking object relative clauses.

### Methods

#### Participants.

We recruited 48 speakers with French as their first language (mean age: 35 years, *SD* = 11) through the Prolific platform (https://www.prolific.co), which compensated the participants at a rate of approximately £9 per hour.

#### Materials and Design.

We employed a 2 × 2 design. The variables manipulated were Verb Type (subject-biased and object-biased verbs) and Relative Clause Type (subject and object relative clauses), resulting in 4 conditions per item.

For the selection of subject-biased and object-biased verbs, we used a corpus of implicit causality biases of French verbs from Mertz et al. (n.d.) (available in the OSF repository here: https://osf.io/53dzc/). On the corpus scale from −100 (object-biased) to 100 (subject-biased) in French, the selected subject-biased verbs had a mean of approximately 73, while the object-biased verbs had a mean of −69.

Each item consisted of a single sentence containing a relative clause. An example of an item for all conditions is provided in Table 1. Each sentence was followed by a comprehension question, which was a partial interrogative focusing on the object of the relative clause. Participants had to choose between the two nouns of the sentence (*Who is worried?*). A total of 20 items were created, with 5 items per condition (following a Latin-Square design). All relative clauses featured an animate subject and an animate object to control for potential animacy effects (Traxler et al., 2005). Additionally, 45 fillers were included (covering independent experiments on active/passive infinitives, subject islands and temporal surbordinate constructions), each followed by a comprehension question.

**Table 1.** Example of one item used in the acceptability judgment task in all conditions

Subject relative	Subject-biased Verb	Le professeur qui affole l’avocat ne donnera plus ce cours au prochain semestre.
		*The teacher that worries the lawyer will not give classes next semester.*
	Object-biased Verb	Le professeur qui choisit l’avocat ne donnera plus ce cours au prochain semestre.
		*The teacher that chooses the lawyer will not give classes next semester.*
Object relative	Subject-biased Verb	Le professeur que l’avocat affole ne donnera plus ce cours au prochain semestre.
		*The teacher that the lawyer worries will not give classes next semester.*
	Object-biased Verb	Le professeur que l’avocat choisit ne donnera plus ce cours au prochain semestre.
		*The teacher that the lawyer chooses will not give classes next semester.*

#### Procedure.

The experiment was conducted using an adapted version of IbexFarm (Drummond, 2013) hosted on university servers. Participants read sentences on a computer screen at a location of their choice. They were instructed to judge the acceptability of each sentence on a scale from 1 (not at all acceptable) to 10 (fully acceptable)^7^, and then answered a comprehension question for each item. The experiment lasted approximately 20 minutes.

### Results

#### Methods of Analysis.

The dependent variables were the ratings for acceptability judgments (from 1 to 10) and the answers to comprehension questions (0 for incorrect answer and 1 for correct answer). We applied mean-centered coding for the independent variables, coding 1 for subject-biased verbs, 0 for object-biased verbs for Verb Type, and 1 for subject relative clauses, 0 for object relative clauses for Clause Type, with subsequent scaling so that the values approached −0.5 for 0 coding and 0.5 for 1 coding. Random variables were Participants and Items. For all random variables, we included random slopes for Verb Bias and Clause Type, as well as their interactions, in accordance with the maximal model justified by the experimental design (Barr et al., 2013).

For the acceptability judgment task, we ran Bayesian ordinal regression models using the cumulative family (ordinal dependent variable). The model was run with 4 MCMC chains and between 3000 and 12000 iterations per chain, depending on the model. As for comprehension questions (binomial dependent variable), we submitted our data to Bayesian binomial regression models using the Bernoulli family. The model was run with 4 MCMC chains and between 3000 and 9000 iterations per chain, depending on the model.

For all analyses in this paper, we ran Bayesian models using the brms package (Bürkner, 2017; Bürkner & Charpentier, 2018; Carpenter et al., 2017), with R version 4.3.3 software (R Core Team, 2023) and the RStudio Pro interface (Posit Team, 2023) on the Huma-Num server. Data and scripts are available in the OSF repository.

Bayesian analyses offer multiple advantages, such as the ability to fit a maximal random effects structure (Barr et al., 2013) without convergence failure, even with relatively small datasets. They also directly test the likelihood of the hypothesis of interest, unlike Frequentist frameworks, and allow us to focus on the probability distribution of the effect rather than a binary decision threshold.

For the interpretation of all Bayesian models, each model generates a posterior distribution for the model parameters of interest (e.g., independent variables). For each analysis in the paper, we report the estimated mean ($\hat{\beta }$), the range (95% credible interval, i.e., the probability that it includes the true value of the parameter), and the probability of the effect of the parameter being smaller than (for negative estimates) or greater than (for positive estimates) zero (P(*β*)). We refer to the probability of the effect (Vasishth, 2023) to estimate the influence of the parameter on the dependent variable. We do not interpret the results as significant or not, but rather focus on the probability distribution of the effect. Following previous studies, the probabilities reported will be interpreted as follows:

▪  if the probability of the effect of the parameter to be different from zero is ≥0.95 and the credible intervals does not include zero, we will interpret this as strong evidence for an effect on the dependent variable;
▪  if the probability of the effect of the parameter to be different from zero is between 0.80 and 0.95, we will interpret this as weak evidence—but still meaningful—for an effect on the dependent variable;
▪  if the probability of the effect of the parameter to be different from zero is less than 0.80, the effect will not be discussed (while still reported in the table).

#### Hypothesis.

If implicit causality modulates relative clause comprehension difficulty, object relative clauses with subject-biased verbs should be less acceptable and comprehension accuracy should be lower than for object relative clauses with object-biased verbs. Effects of verb bias are not necessarily expected for subject relative clauses; however, if such effects are present, they should manifest in the opposite manner, with subject relative clauses with object-biased verbs being less acceptable compared to those with subject-biased verbs.

#### Acceptability Judgments.

**Figure 1.**
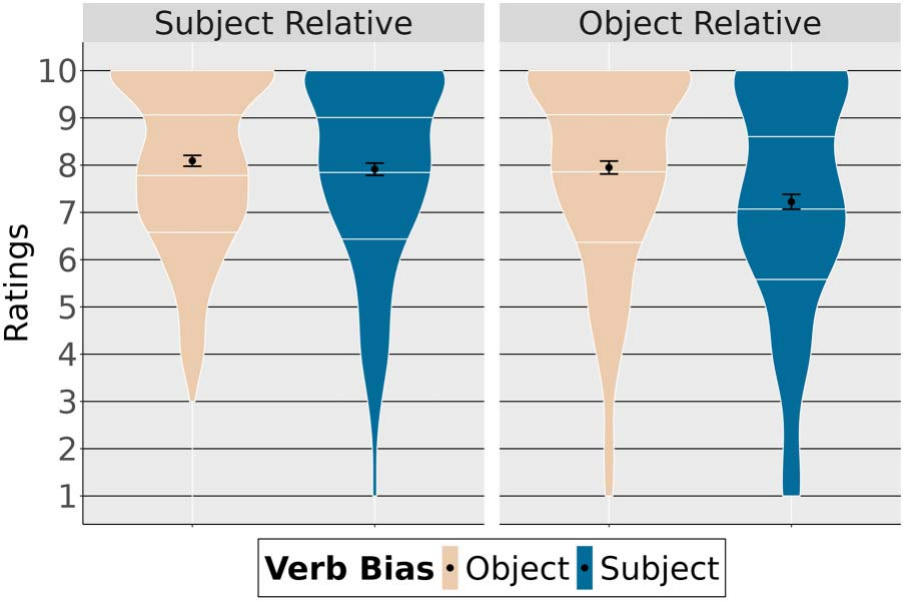
Ratings depending on relative clause type and verb bias.

Violin plots in Figure 1 show the mean acceptability judgments depending on relative clause type and relative clause verb bias. They also show the quartiles (white horizontal lines) giving more detailed information about the distribution of the data.

Table 2 summarizes the fixed effects. Given the data, we found that ratings were lower with subject-biased verbs than with object-biased verbs (high probability of an effect of verb bias: P(*β* < 0) = 1). Subject relative clauses were generally more acceptable than object relative clauses (high probability of an effect of clause type: P(*β* > 0 = 0.99). Finally, verb bias plays a different role depending on the type of relative clause (high probability of an interaction between the two factors: P(*β* > 0) = 0.98), indicating that for object relative clauses, sentences with subject-biased verbs are judged less acceptable than sentences with object-biased verbs compared to subject relative clauses.

**Table 2.** Results from the ordinal model (acceptability judgment)

	Effect	$\hat{\beta }$	**P**(*β* > 0)	**P**(*β* < 0)	**95% CrI**
*Main Analysis*	Verb Bias	−0.54		1	[−0.91, −0.17]
	Clause Type	0.36	0.99		[0.06, 0.67]
	Verb Bias:Clause Type	0.70	0.98		[0.02, 1.39]
*Subject Relatives*	Verb Bias	−0.19		0.80	[−0.64, 0.27]
*Object Relatives*	Verb Bias	−0.87		1	[−1.42, −0.31]

#### Comprehension Questions.

**Figure 2.**
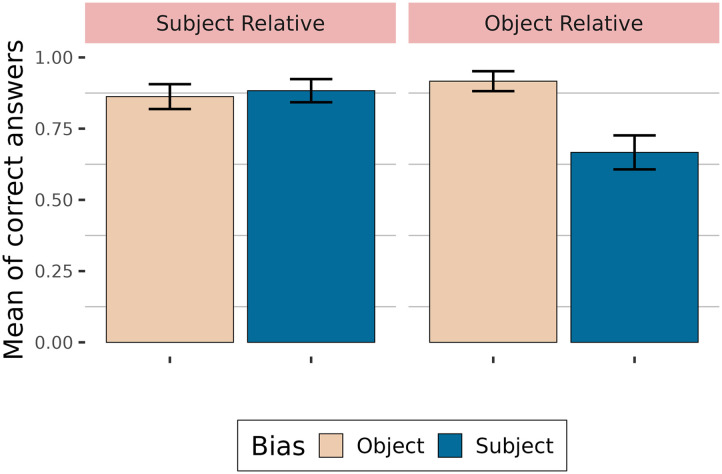
Accuracy depending on relative clause type and verb bias.

Proportions of correct answers to comprehension questions are shown in Figure 2. Participants answered correctly more than 70 % of the time.

Table 3 summarises the fixed effects. The results showed that relative clauses with subject-biased verbs are less well understood than relative clauses with object-biased verbs in general (high probability of an effect of verb bias: P(*β* < 0) = 1). Subject relative clauses appear to be better understood than object relative clauses (moderate probability of an effect: P(*β* > 0) = 0.94). Additionally, verb bias does not play the same role in subject relative clauses as in object relative clauses (high probability of an interaction: P(*β* > 0) = 1), with answers to questions with subject-biased verbs being less accurate in object relative clauses than for questions with object-biased verbs, compared to subject relative clauses.

**Table 3.** Results from the logistic model (accuracy)

	Effect	$\hat{\beta }$	**P**(*β* > 0)	**P**(*β* < 0)	**95% CrI**
*Main Analysis*	Verb Bias	−0.96		1	[−1.64, −0.34]
	Clause Type	0.58	0.94		[−0.16, 1.37]
	Verb Bias:Clause Type	2.63	1		[1.33, 4.13]
*Subject Relatives*	Verb Bias	0.38	0.78	0.22	[−0.55, 1.48]
*Object Relatives*	Verb Bias	−2.13		1	[−3.25, −1.18]

### Discussion

Results from acceptability judgment data and from comprehension questions in French showed that object relative clauses with subject-biased verbs were least acceptable.

Additionally, the interaction between verb bias and clause type we found showed that verb bias had a stronger effect in object relative clauses than in subject relative clauses, and it particularly affected object relative clause acceptability, thus contributing to the main effect of relative clause type.

In general, our results are fully compatible with the hypothesis that verb bias plays a more important role for object relative clauses, impeding comprehension and acceptability when there is a conflict between the subject of the object relative clause and the antecedent, i.e., in the case of subject-biased verbs. The lesser effect in subject relative clauses can be explained by the fact that there is no interference from any factors, allowing the antecedent to be the topic of the relative clause. Subjects inherently are likely to be aboutness topics (as well as antecedents). In the case of a subject relative clause, both requirements are fulfilled such that verb bias does not exert an equally strong effect. Surprisingly, the effect of verb bias seems to go in the opposite direction than we predicted for subject-relative clauses. One explanation could be frequency: we found that subject-biased verbs are generally less frequent than object-biased verbs for the verbs we used in our experiment (we will come back to this in Experiment 2).

However, this experiment only provides comprehension and acceptability data at the final stages of processing. We cannot conclude from our data whether or not the initial processing cost is affected by implicit causality biases. Moreover, we did not control for the frequency of the different types of verbs in Experiment 1. In Experiment 2, we investigated to what extent implicit causality directly influences relative clause processing through a self-paced reading task.

## EXPERIMENT 2

Experiment 2 is an extension of Experiment 1 in that we collected phrase by phrase self-paced reading data as well as comprehension data and we controlled for potential frequency effects.

### Methods

#### Participants.

We recruited 40 speakers with French as their first language (mean age: 34 years old, *SD* = 10) via the Prolific platform, which compensated participants at a rate of approximately £9 per hour.

#### Materials and Design.

The variables manipulated were Verb Type (subject-biased and object-biased verbs) and Relative Clause (subject and object relative clauses), resulting in 4 conditions per item. We modified the sentences from the previous experiment to better calibrate them for the self-paced reading task. We created 20 items with 5 items per condition (an example is given in Table 4). For verb selection, we used the same corpus as in the first experiment, but this time we controlled for verb frequency. We aimed to have similar frequencies between the two verbs used for each item (see the OSF repository), with an average lemma frequency of 55 (out of a million words) for subject-biased verbs and 52 for object-biased verbs. The frequency was taken from the database of New et al. (2004). More specifically, we used the frequency of the verb lemma from the corpus of subtitles (per million of occurrences). Each pair of verbs was used for two different items, allowing participants to see both the subject-biased verb and the object-biased verb in different items.

**Table 4.** Example of two items used in the self-paced reading task in all conditions

		NP	rel	rel+1	rel+2	post-rel	post-rel+1	post-rel+2
SR	Subject-biased Verb	Le professeur	qui	affole	le directeur	mange	au restaurant	le midi.
		*The teacher*	*that*	*worries*	*the director*	*eats*	*in the restaurant*	*for lunch.*
	Object-biased Verb	Le professeur	qui	redoute	le directeur	mange	au restaurant	le midi.
		*The teacher*	*that*	*fears*	*the director*	*eats*	*in the restaurant*	*for lunch.*
OR	Subject-biased Verb	Le professeur	que	le directeur	affole	mange	au restaurant	le midi.
		*The teacher*	*that*	*the director*	*worries*	*eats*	*in the restaurant*	*for lunch.*
	Object-biased Verb	Le professeur	que	le directeur	redoute	mange	au restaurant	le midi.
		*The teacher*	*that*	*the director*	*fears*	*eats*	*in the restaurant*	*for lunch.*

Prior to the experiment, we ran a plausibility norming study for the relative clauses (with different participants) to ensure that the combination of the verb and the NPs was plausible (e.g., *The teacher worries the lawyer* vs. *The lawyer worries the teacher*). We found that participants judged the sentences as highly plausible (mean: 0.89/1). To control for possible remaining semantic biases, we created two versions of each item. In the second version, we exchanged the referents occupying the roles of subject and object so that the object of the relative clause (*The teacher that worries the lawyer*) became the subject in the other version (*The lawyer that worries the teacher*), for both subject and object relative clauses. We thus created two counterbalanced versions of the experiment and, for the statistical analysis, we considered both versions of each item as one unique item.

After each sentence, there was a comprehension question. In this experiment, the question focused on the subject or the object of the relative clause, and participants had to choose between the two nouns of the sentence or between yes and no. Additionally, 42 fillers were added, consisting of simple sentences and other subordinate constructions.

#### Procedure.

The experiment was conducted using an adapted version of IbexFarm (Drummond, 2013) hosted on university servers. Participants read sentences that appeared one segment at a time in a moving window non-cumulative paradigm on a computer screen at a location of their choice. They were instructed to carefully read the sentences at their own pace, pressing the space bar to reveal each subsequent word. After reading the sentence, they had to answer a comprehension question by choosing between two possible answers.

### Results

#### Method of Analysis.

The dependent variables were reading times and answers to comprehension questions (0 for incorrect answers and 1 for correct answers). Reading times were analysed using linear-mixed models with a lognormal likelihood. The independent variables were coded as follows: 1 for subject-biased verb and 0 for object-biased verb for Verb Type, and 1 for subject relative and 0 for object relative. We applied scaled mean-centered coding such that 0 coding approached −0.5 and 1 coding approached 0.5. The model took into account the length of the region analyzed (whenever relevant). For all random variables, we included random slopes for Verb Bias and Clause Type, as well as their interactions, in accordance with the maximal model justified by the experimental design (Barr et al., 2013). As for the outliers for reading times, inspired by Paape and Vasishth (2022), trials for which reading time was longer than 5,000 ms were removed (0.02% of the data), and those for which reading time was shorter than 150 ms were also removed (0.63% of the data).

#### Hypothesis.

If object relative clauses with subject-biased verbs are more difficult to process than those with object-biased verbs, we will expect higher reading times in the region after the relative clause, specifically at the main clause verb (post-rel). We do not expect any strong difference in reading times between subject-biased verbs and object-biased verbs in subject relative clauses in these regions.

#### Reading Times.

**Figure 3.**
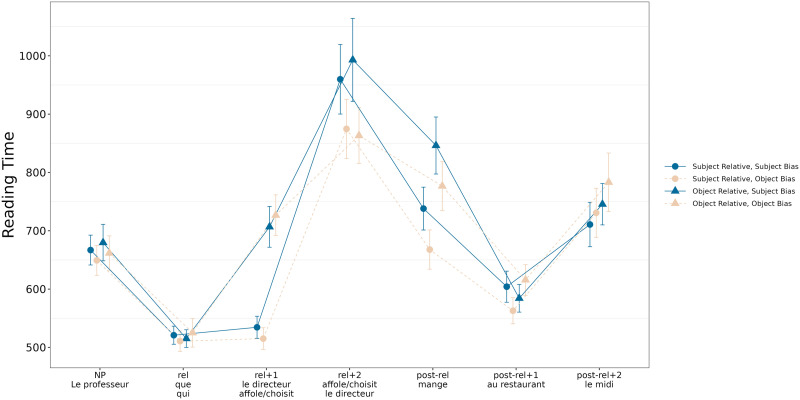
Reading times for subject and object relative clauses depending on verb bias (ms).

Figure 3 represents the reading times of all regions in the sentence depending on relative type (subject or object) and verb bias (subject or object).

We report results from the relative clause (rel+1: subject in object relative clauses and verb in subject relative clauses, as well as rel+2: verb in object relative clauses and the object in subject relative clauses), and from the the main clause verb (the region thereafter, post-rel) since this region is parallel across conditions.

In the beginning of the relative clause, at rel+1 (cf. Table 5), given the data, there was a moderate probability of an effect of clause type, indicating that subject relative clauses seemed to be read faster than object relative clauses (P(*β* < 0) = 0.87). We also found a moderate probability of an interaction between clause type and verb bias (P(*β* > 0) = 0.86), with subject-biased verbs being read more slowly than object-biased verbs in subject relative clauses especially. Finally, we found a high probability of an effect of length, indicating that reading times increased when the word was longer (P(*β* > 0) = 1).

**Table 5.** Results from the model (reading time) rel+1

	Effect	$\hat{\beta }$	**P**(*β* > 0)	**P**(*β* < 0)	**CrI**
*Main Analysis*	Length	0.03	1		[0.02, 0.05]
	Verb Bias	0.01	0.68	0.32	[−0.04, 0.07]
	Clause Type	−0.07		0.87	[−0.19, 0.05]
	Verb Bias:Clause Type	0.06	0.86		[−0.05, 0.17]
*Subject Relatives*	Verb Bias	0.04	0.89		[−0.03, 0.10]
*Object Relatives*	Verb Bias	−0.02	0.37	0.63	[−0.11, 0.08]

At the end of the relative at rel+2 (cf. Table 6), we found that relative clauses with subject-biased verbs were read more slowly than with object-biased verbs (high probability of an effect, P(*β* > 0) = 0.95).

**Table 6.** Results from the model (reading time) rel+2

Effect	$\hat{\beta }$	**P**(*β* > 0)	**P**(*β* < 0)	**CrI**
Length	0.001	0.54	0.46	[−0.02, 0.02]
Verb Bias	0.07	0.95		[−0.02, 0.15]
Clause Type	−0.02	0.39	0.61	[−0.17, 0.13]
Verb Bias:Clause Type	−0.005	0.48	0.52	[−0.16, 0.15]

At the main clause verb (post-rel region, Table 7), we found that relative clauses with subject-biased verbs had longer reading times than relative clauses with object-biased verbs, with a high probability of an effect (P(*β* > 0) = 0.98). Subject relative clauses were also read faster than object relative clauses (high probability of an effect of clause type, P(*β* < 0) = 0.97). Finally, we found a high probability of an effect of length, indicating that reading times increased when the verb was longer (P(*β* > 0) = 0.98).

**Table 7.** Results from the model (reading time) at post-rel

Effect	$\hat{\beta }$	**P**(*β* > 0)	**P**(*β* < 0)	**95% CrI**
Length	0.04	0.98		[0.003, 0.08]
Verb Bias	0.09	0.98		[0.01, 0.17]
Clause Type	−0.10		0.97	[−0.19, 0.003]
Verb Bias:Clause Type	0.03	0.67	0.33	[−0.12, 0.18]

#### Comprehension Questions.

**Figure 4.**
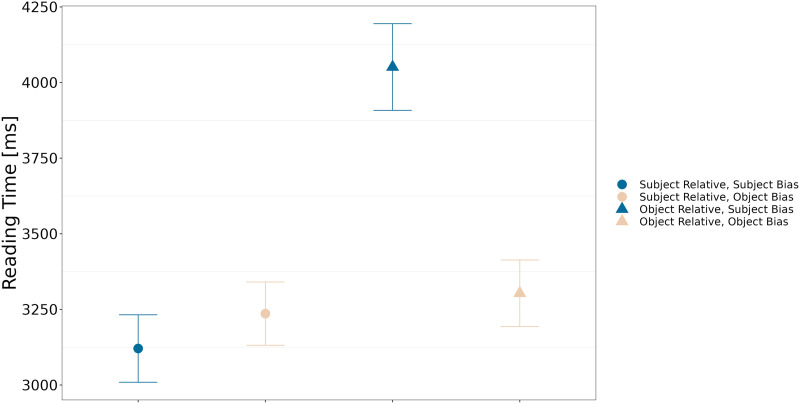
Answering times for questions depending on relative clause type and verb bias.

**Figure 5.**
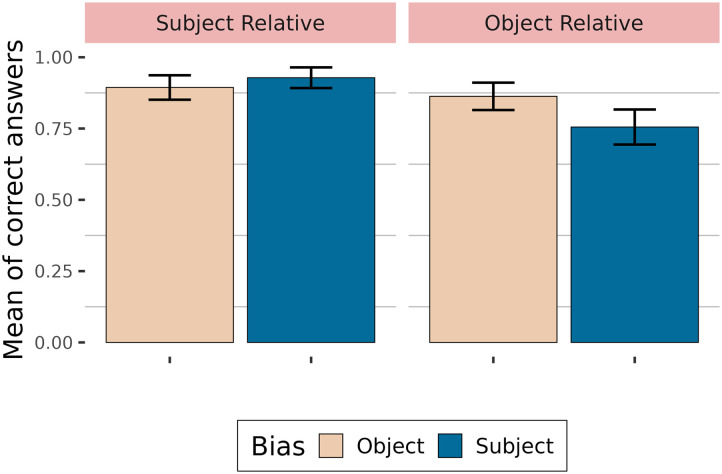
Accuracy depending on relative clause type and verb bias.

Figure 4 and Figure 5 respectively represent the mean answering times for questions and the mean accuracy depending on relative clause type (subject or object) and verb bias (subject or object). Trials for which reading time was longer than 10,000 ms were removed (2.75% of the data).

For mean answering times (cf. Table 8), we found that questions on sentences with subject-biased verbs took more time to be answered than questions on sentences with object-biased verbs, with a high probability of an effect (P(*β* > 0) = 0.98). Answers to subject relative clauses were faster (high probability of an effect of clause type, P(*β* < 0) = 1). Additionally, the difference between subject- and object-biased verbs was stronger in object relative clauses than in subject relative clauses (high probability of an interaction between the two factors, P(*β* < 0) = 1).

**Table 8.** Results from the model (time answering)

	Effect	$\hat{\beta }$	**P**(*β* > 0)	**P**(*β* < 0)	**CrI**
*Main Analysis*	Verb Bias	0.07	0.98		[0.01, 0.14]
	Clause Type	−0.13		1	[−0.22, −0.05]
	Verb Bias:Clause Type	−0.25		1	[−0.39, −0.11]
*Subject Relatives*	Verb Bias	−0.05		0.87	[−0.14, 0.04]
*Object Relatives*	Verb Bias	0.20	1		[0.11, 0.29]

For accuracy in Table 9, we found that sentences with a subject relative clause were better understood than sentences with an object relative clause (high probability of an effect: P(*β* > 0) = 0.98). Also, the difference in accuracy between relative clauses with subject-biased verbs and those with object-biased verbs was stronger for object relative clauses than for subject relative clauses (high probability of an interaction between the two factors: P(*β* > 0) = 0.98).

**Table 9.** Results from the logistic model (accuracy)

	Effect	$\hat{\beta }$	**P**(*β* > 0)	**P**(*β* < 0)	**CrI**
*Main Analysis*	Verb Bias	0.01	0.49	0.51	[−0.80, 0.91]
	Clause Type	0.99	0.98		[0.05, 1.97]
	Verb Bias:Clause Type	1.57	0.98		[0.15, 3.13]
*Subject Relatives*	Verb Bias	0.80	0.90		[−0.42, 2.27]
*Object Relatives*	Verb Bias	−0.70		0.92	[−1.68, 0.34]

### Discussion

We expected higher reading times for sentences with subject-biased verbs compared to sentences with object-biased verbs in object relative clauses only. We did not expect verb bias to affect reading times in subject relative clauses, but if so, object-biased verbs should make understanding harder than subject-biased verbs in this construction. Surprisingly, our results showed that object-biased verbs led to faster reading times for subject as well as for object relative clauses (as it seemed to be the case for acceptability judgments in Experiment 1). However, this general advantage for object-biased verbs in processing did not affect comprehension as measured in accuracy and question answering times where object-biased verbs led to better comprehension in object relative clauses only. Notably, our results are compatible with Gennari and MacDonald (2008) who report thematic role effects for object relative clauses similar to those we found. However, unlike our study, Gennari and MacDonald (2008) did not include subject relative clauses in their design and, more importantly, they did not consider the implicit causality of the verb as a factor.

The unexpected effect of verb bias in subject relative clauses requires further explanation. One possible explanation could be type frequency (class of verbs) and not token frequency (lemma frequency). Even though we controlled for the effect of token frequency in our experiment, as well as the relative clause plausibility (for subject and object relative clauses), subject-biased verbs are generally less frequent than object-biased verbs. Two reasons can explain the results. From a usage-based perspective, type—rather than token—frequency is crucial to the productivity of syntactic patterns (Bybee, 1995; Bybee & Thompson, 1997). It is possible that participants may have had difficulties with a subject-biased verb because they did not expect to have the causer of the event (= the verb) attributed to the subject, but rather to the object. The longer reading times for subject-biased verbs may be due to the unexpectedness of the verb, which can be interpreted as a surprisal effect (Hale, 2001). Second, in this experiment, we did not consider the thematic roles of the verbs, which may have affected the results: subject-biased verbs often are stimuli-experiencer verbs (see Experiment 4 for a more detailed discussion).

As for early processing, our results differ for reading times compared to accuracy and acceptability judgments. It seems that the integration of implicit causality occurs at a rather late stage of processing. Related to this, Caplan and Waters (1999) suggested two levels of processing: interpretative processing and post-interpretative processing. Interpretative processing would be devoted to linguistic tasks such as word recognition, syntactic and prosodic representations and some discourse-level semantics like thematic role assignation. Post-interpretative processing would refer to other rather less automatic functions such as reasoning, or storing information in long-term semantic memory (Caplan & Waters, 1999). Looking back at our results, it appears that the interaction of verb bias with clause type may at least partly play out at a post-interpretative processing stage, especially when participants are asked to judge a sentence or need to remember the sentence when answering a comprehension question.

Another possibility to explain the rather late integration of implicit causality during processing can be related to a good-enough processing (Ferreira et al., 2002). In the good-enough approach, language processing is partial and semantic representations are shallow. From this perspective, participants process sentences until achieving a satisfying representation, even if it is incomplete. In this case, implicit causality would not be entirely interpreted at a first stage due to good-enough processing, but only when we ask for judgment on the complete interpretation that it is integrated into the semantic representation of the sentence.

So far, our results suggest that implicit causality interferes in relative clause processing, though only at later stages of processing. However, our results could also be due to other factors than implicit causality that we did not control. This is what we are going to test in the next two experiments, namely the effect of the intervening subject in the relative clause as well the effect of thematic roles.

## EXPERIMENT 3

Our previous experiments demonstrated an effect of implicit causality on relative clause comprehension. However, it is possible that our results could also be explained by other syntactic and semantic factors that we did not manipulate.

An alternative account of our data would be compatible with theories that stress the importance of an intervening subject such as the Relativized Minimality (Rizzi, 1990), the Dependency Locality Theory (Gibson, 2001), or the similarity-based interference theory (Gordon et al., 2006). They claim, from a syntactic or, respectively, a memory-based standpoint, that the position of the subject in the relative clause plays a role in processing. In this case, implicit causality would be *a secondary factor*, possibly making reanalysis easier.

Different from English, French object relative clauses can have a preverbal subject (21) or a postverbal subject (22)^8^ (Kayne & Pollock, 1978; Le Bidois, 1952). The postverbal subject does not linearly intervene between the object and the gap, as in (24), even though it may still structurally intervene, see (23).

(21)  Object Relative with preverbal subject
    L’avocat [_CP_ que le professeur connait]
    The lawyer [_CP_ that_*obj*_ the teacher knows]
    ‘The lawyer that the teacher knows’
(22)  Object Relative with postverbal subject
    L’avocat [_CP_ que connait le professeur]
    The lawyer [_CP_ that_*obj*_ knows the teacher]
    ‘The lawyer that the teacher knows’
(23)  Simplified structural representation of object relative, postverbal subject
    
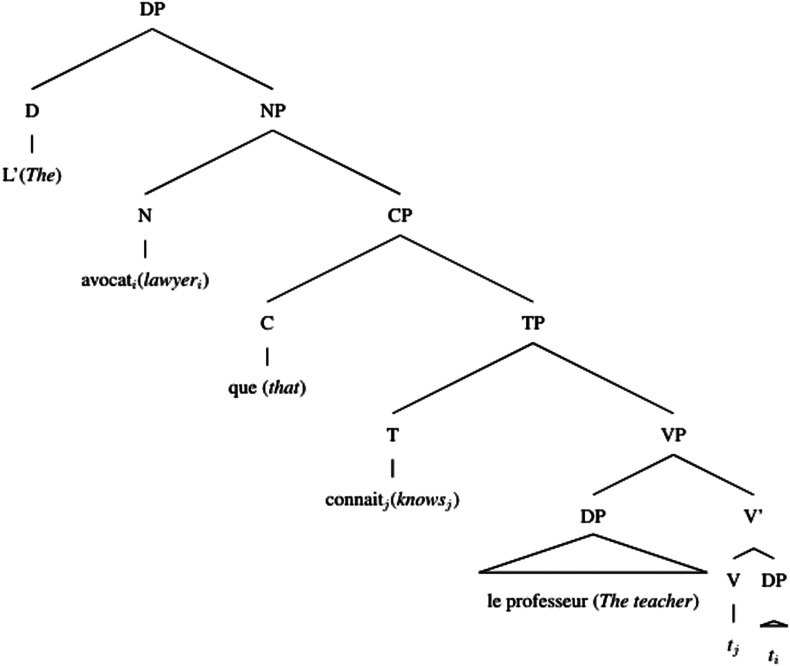
(24)  Linear representation of object relative, postverbal subject
    
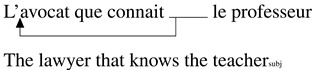

Depending on the syntactic theory, there is less (or no) subject intervention between the gap and the filler when the subject is postverbal. Therefore, smaller effects of implicit causality might be expected in object relative clauses with postverbal subjects compared to object relative clauses with preverbal subjects if implicit causality is merely a secondary factor.

From a memory-based point of view, linear distance makes different predictions based on the position of the subject: object relative clauses with postverbal subjects should be easier to process than object relative clauses with preverbal subjects because of the shorter linear distance between the object and the gap as well as the non-intervention of the subject (see (24)). Regarding similarity-based interference theories (Gordon et al., 2006), since the subject appears after the integration of the antecedent with the verb in object relative clauses with postverbal subjects, processing should be easier compared to object relative clauses with preverbal subjects.

Concretely, if the intervening subject is the primary factor explaining reduced acceptability of relative clauses, and if verb bias *only* modulates this primary factor, there would be a difference in acceptability and accuracy between object relative clauses with preverbal subjects and object relative clauses with postverbal subjects regarding the verb bias condition. Indeed, contrary to object relative clauses with preverbal subjects, object relative clauses with postverbal subjects should be less (or even not at all) influenced by verb bias since the subject intervenes to a lesser extent in this configuration^9^.

To clarify these questions, we conducted an acceptability judgment task for object relative clauses, manipulating only the position of the subject.

### Methods

#### Participants.

We recruited 39 speakers with French as their first language (mean age: 41 years old, *SD* = 18) on the RISC platform (https://www.risc.cnrs.fr).

#### Materials and Design.

We employed a 2 × 2 design with the same materials as in the first experiment, with the exception that we manipulated the position of the subject in object relative clauses (preverbal and postverbal subjects) instead of the type of relative clause (subject and object relative clauses). An example of an item is shown in Table 10 for all conditions. Additionally, 39 other fillers were added from an independent experiment on coordination (with comprehension questions after each sentence).

**Table 10.** Example of one item used in the acceptability judgment task in all conditions

Preverbal subject	Subject-biased Verb	Le professeur que l’avocat affole ne donnera plus ce cours au prochain semestre.
		*The teacher that the lawyer worries will not give classes next semester.*
	Object-biased Verb	Le professeur que l’avocat choisit ne donnera plus ce cours au prochain semestre.
		*The teacher that the lawyer chooses will not give classes next semester.*
Postverbal subject	Subject-biased Verb	Le professeur qu’affole l’avocat ne donnera plus ce cours au prochain semestre.
		*The teacher that_obj_ worries the lawyer will not give classes next semester.*
	Object-biased Verb	Le professeur que choisit l’avocat ne donnera plus ce cours au prochain semestre.
		*The teacher that_obj_ chooses the lawyer will not give classes next semester.*

#### Procedure.

The procedure was the same as in the acceptability judgment task in Experiment 1.

### Results

#### Methods of Analysis.

Dependent variables were the ratings for acceptability judgments (from 0 to 10) and answers to comprehension questions (0 for incorrect answers and 1 for correct answers). The independent variables were coded as follows: 1 for subject-biased verb and 0 for object-biased verb for Verb Type, and 1 for object relative with postverbal subject and 0 for object relative with preverbal subject for Subject Position. We applied mean-centered coding by scaling the coded variables such that 0 coding approached −0.5 and 1 coding approached 0.5. For all random variables, we included random slopes for Verb Bias and Clause Type, as well as their interactions, in accordance with the maximal model justified by the experimental design (Barr et al., 2013).

#### Hypothesis.

If implicit causality enhances effects of subject intervention, verb bias should not play the same role in object relative clauses with preverbal subjects as with with postverbal subjects: relative clauses with preverbal subjects should be less acceptable (and comprehension questions less accurate) with subject-biased verbs compared to object relative clauses with postverbal subjects. But if implicit causality interferes with the topicworthiness of the antecedent, object relative clauses with subject-biased verbs should be less acceptable (and comprehension questions less accurate) independent of subject position.

#### Acceptability Judgments.

**Figure 6.**
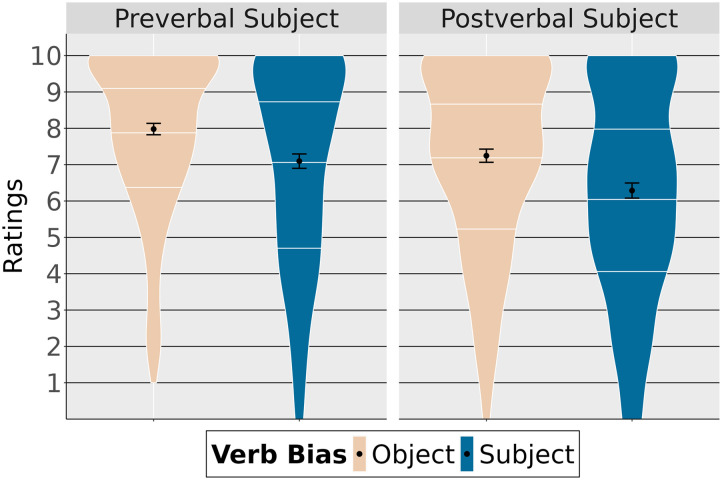
Ratings depending on subject position and verb bias.

Violin plots in Figure 6 show the mean acceptability judgments depending on the position of the subject (preverbal/postverbal) and relative clause verb bias (subject/object) as well as quartiles indicating the distribution of the data. Object relative clauses with subject bias and postverbal subjects were judged least acceptable.

Table 11 summarizes the fixed effects. Subject-biased verbs were found to be less acceptable than object-biased verbs in general (high probability of an effect of verb bias (P(*β* < 0) = 1). Ratings were also lower for object relative clauses with postverbal subjects than with preverbal subjects (high probability of an effect of the subject position in the relative clause, P(*β* < 0) = 1).

**Table 11.** Results from the ordinal model (acceptability judgment)

	Effect	$\hat{\beta }$	**P**(*β* > 0)	**P**(*β* < 0)	**CrI**
*Main Analysis*	Verb Bias	−1.07		1	[−1.45, −0.69]
	Subject Position	−0.98		1	[−1.43, −0.55]
	Verb Bias:Subject Position	−0.09	0.39	0.61	[−0.73, 0.55]

#### Comprehension Questions.

**Figure 7.**
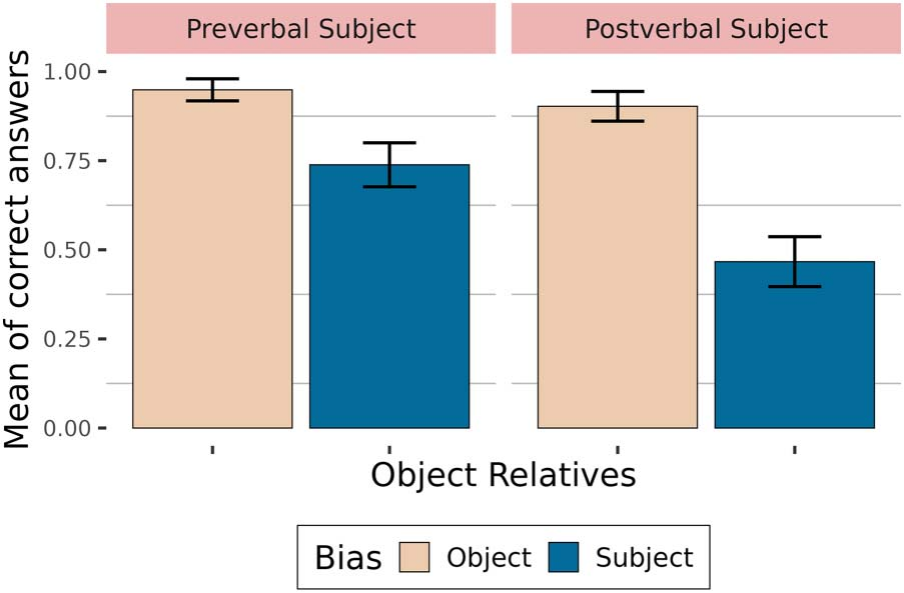
Accuracy depending subject position and verb bias.

Proportions of correct answers to comprehension questions are shown in Figure 7. Again, participants’ answers are well above the level of chance except for object relative clauses with postverbal subjects and subject-biased verbs, which appear to be less well understood.

As confirmed in Table 12, accuracy with subject-biased verbs was reduced compared to accuracy with object-biased verbs (high probability of an effect of verb bias, P(*β* < 0) = 1). We also found that questions about relative clauses with postverbal subjects were less accurate than those about relative clauses with preverbal subjects (high probability of an effect of subject position, P(*β* < 0) = 0.99). Finally, the decrease in accuracy for subject-biased verbs was larger for object relative clauses with postverbal subjects than with preverbal subjects (high probability of an interaction, P(*β* < 0) = 0.96).

**Table 12.** Results from the logistic model (accuracy)

	Effect	$\hat{\beta }$	**P**(*β* > 0)	**P**(*β* < 0)	**CrI**
*Main Analysis*	Verb Bias	−3.01		1	[−4.12, −2.08]
	Subject Position	−1.08		0.99	[−1.95, −0.16]
	Verb Bias:Subject Position	−1.49		0.96	[−3.35, 0.24]
*Preverbal Subject*	Verb Bias	−2.00		1	[−3.41, −0.77]
*Postverbal Subject*	Verb Bias	−4.10		1	[−5.97, −2.75]

### Discussion

Object relative clauses with postverbal subjects were overall less well understood and judged less acceptable than those with preverbal subjects. Pozniak et al. (2021) found that object relative clauses with postverbal subjects can be as acceptable as object relative clauses with preverbal subjects depending on different properties (when the subject is long and non-agentive for example). In our experiments, subjects were always agentive and relatively short, which can explain why object relative clauses with postverbal subjects were disfavored.

Compared to the first acceptability judgment task (Experiment 1), the effect of verb bias in object relative clauses with preverbal subjects was replicated: object relative clauses with object-biased verbs were better understood and judged more acceptable than object relative clauses with subject-biased verbs. The hypothesis that implicit causality effects play only a secondary role for linearly intervening subjects would have predicted an interaction between subject position and verb bias, such that the verb bias effect should be stronger for object relative clauses with preverbal subjects. However, in the comprehension questions, there was an interaction between verb bias and subject position going in the opposite direction, with verb bias playing a stronger role in relative clauses with postverbal subjects than in those with preverbal subjects. The predicted interaction did not appear in acceptability judgments either: no reliable interaction between verb bias and subject position in acceptability judgments was found. The effect of verb bias was the same in both types of object relative clauses independent of the position of the subject, meaning that relative clauses with postverbal subjects were less understood and judged less acceptable when the relative clause verb was subject-biased. All this suggests that the role of verb bias is not merely a secondary factor in our experiments and actually may interfere with the topicworthiness of the antecedent. Our results may still be compatible with a structural distance hypothesis in an analysis as in Figure (23) where the subject structurally intervenes between the antecedent and the gap. However, the acceptability differences cannot be explained by syntactic factors alone.

The difference in accuracy for implicit causality biases depending on the position of the subject in the relative clause may be explained by internal subject properties. As said before, Pozniak et al. (2021) found that—among other factors—a subject will be more likely to be postverbal when it is non agentive and non intentional whereas a preverbal subject will be preferred with agentive properties. Having an agentive subject in the postverbal position will then make the object relative clause less acceptable. Moreover, a subject-biased verb looks for a subject with agentive properties (for example, the verb *trouble* will rather look for an animate subject that is the cause of the event). Also, subject-biased verbs are likely to consider the subject as the aboutness topic. However, it has been claimed that postverbal subjects are detopicalized (Kampers-Manhe et al., 2004; Marandin, 2011). Hence, postverbal subjects may be less compatible with subject-biased verbs. As a consequence, speakers may be inclined to assume that a subject relative clause was intended here, only replacing the *que* with *qui* as suggested by noisy channel models (Gibson et al., 2013).

It is important to mention that we did not explicitly manipulate thematic roles in our three experiments, while it has been found that thematic roles, interacting with animacy, also play a role in object relative clause processing. As mentioned before, Gennari and MacDonald (2009) found that object relative clauses with stimulus-experiencer verbs were more difficult to process than object relative clauses with agent-patient verbs in English. However, when looking at the verbs used in Gennari and MacDonald (2009), we found that implicit causality was not controlled for: in the stimulus-experiencer verb list, most verbs were subject-biased according to Ferstl et al. (2011) (a mean around 67 for 23 verbs with 100 meaning fully subject-biased and −100 fully object-biased), which could explain the processing difficulty^10^.

In order to resolve this potential confound and to find out whether our results may be due to thematic roles, we ran another acceptability judgment experiment manipulating the thematic role of the verb.

## EXPERIMENT 4

As mentioned in the introduction, relative clause processing is modulated by factors such as animacy of the nouns (Frauenfelder et al., 1980; Gennari & MacDonald, 2008; Mak et al., 2002) but also thematic roles attributed by the verb (Gennari & MacDonald, 2009). Indeed, Gennari and MacDonald (2009) showed in their Experiments 5 and 6 that object relative clauses will be harder to process with stimulus-experiencer verbs (theme-experiencer verbs in Gennari & MacDonald, 2009) than with agent-patient verbs^11^. However, when looking at the verbs used in the experiments in Gennari and MacDonald (2009), most stimulus-experiencer verbs were also subject-biased according to Ferstl et al. (2011), while this did not seem to be the case for agent-patient verbs. This is to be expected since, as explained in Gennari and MacDonald (2009), a verb will be stimulus-experiencer when someone or something—the stimulus—causes a change in someone—the experiencer. A subject bias is thus part of the definition of a stimulus-experiencer verb.

For example, with a verb like *delight* (taken from Ferstl et al., 2011), the subject will cause a change in the object: in (25), Mary (the subject) changed the emotional state of Hannah (the object) such that Hannah feels delighted. The change that Hannah experiences can only be explained by providing more information about the causer of the event, the stimulus subject *Mary*, which makes the verb *delight* a subject-biased verb. Subject-biased verbs are more likely to come with stimulus-experiencer roles. For agent-patient verbs as in (26), Mary, as an agent, intentionally causes the event but the reason for the event is more likely to be found with Hannah the object, making the verb object-biased (also taken from Ferstl et al., 2011)^12^. In this sense, implicit causality seems to be correlated with thematic roles. We will come back to this in the General Discussion.

(25) 
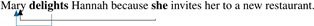
(26) 
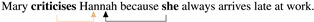

In our previous experiments, we did not control for possible thematic role effects. The logic behind our reasoning was that if implicit causality is part of the definition of thematic roles, it will be nearly impossible to disentangle the two factors. Following Bott and Solstad (2014), it is however possible that implicit causality does not affect processing the same way depending on the thematic roles (see discussion below). This is why we included thematic roles as a factor in another acceptability judgment experiment. We included agent-patient verbs with subject and object bias in this experiment as well as so-called psychological verbs, i.e., subject-biased stimulus-experiencer verbs and object-biased experiencer-stimulus verbs (see Table 13).

**Table 13.** Example of two items used in the acceptability judgment task in all conditions

Subject relative with agent-patient verb	Subject-biased Verb	Le fleuriste qui contacte le parfumeur mange un sandwich pour le midi.
		*The florist who contacts the perfumer eats a sandwich for lunch.*
	Object-biased Verb	Le fleuriste qui reprimande le parfumeur mange un sandwich pour le midi.
		*The florist who berates the perfumer eats a sandwich for lunch.*
Subject relative with psychological verb	Subject-biased Verb	Le policier qui amuse le responsable habite dans un appartement près de la mairie.
		*The policeman who amuses the manager lives in a flat near the town hall.*
	Object-biased Verb	Le policier qui admire le responsable habite dans un appartement près de la mairie.
		*The policeman who admires the manager lives in a flat near the town hall.*
Object relative with agent-patient verb	Subject-biased Verb	Le fleuriste que le parfumeur contacte mange un sandwich pour le midi.
		*The florist who the perfumer contacts eats a sandwich for lunch.*
	Object-biased Verb	Le fleuriste que le parfumeur reprimande mange un sandwich pour le midi.
		*The florist who the perfumer berates eats a sandwich for lunch.*
Object relative with psychological verb	Subject-biased Verb	Le policier que le responsable amuse habite dans un appartement près de la mairie.
		*The policeman who the manager amuses lives in a flat near the town hall.*
	Object-biased Verb	Le policier que le responsable admire habite dans un appartement près de la mairie.
		*The policeman who the manager admires lives in a flat near the town hall.*

### Methods

#### Participants.

Forty-eight speakers with French as their first language (mean age: 34 years old, *σ* = 11) were recruited through the Prolific platform, which compensated participants at a rate of approximately £9 per hour.

#### Materials and Design.

We manipulated the verb bias in subject and object relative clauses, with subject- or object-biased verbs, including thematic roles as a between-item factor (12 agent-patient items, 12 items with psychological verbs). We created 24 items with 3 items per condition. All verbs in the relative clause were taken from the Mertz et al. (n.d.) corpus. Bias in the corpus is coded from −100 (strong object bias) to 100 (strong subject bias). We tried to control for frequency as much as possible with an average lemma frequency of 32 (out of a million words) for object-biased agent-patient verbs, 62 for subject-biased agent-patient words, 37 for object-biased experiencer-stimulus verbs and 40 for subject-biased stimulus-experiencer verbs (all frequencies were taken from the book corpus of Lexique 3.83; New et al., 2004). Because of the thematic role constraint (as well as other constraints like the length of the verb and the frequency in each pair), we had to compromise on the strength of the implicit causality bias for subject and object-biased verbs which were on average 31 and −77 for agent-patient verbs, and 75 and −65 for stimulus-experiencer and experiencer-stimulus verbs (we will come back to this in the discussion). Subject-biased agent-patient verbs are very difficult to find. As a consequence, the bias difference is somewhat bigger for psychological verbs (140) than for agent-patient verbs (108). An example of two items (with agent-patient verbs and stimulus-experiencer/experiencer-stimulus verbs) is shown in Table 13.

Items were constructed similar to the previous experiments, consisting of a single sentence with a relative clause modifying the main clause subject. As in previous experiments, we included a comprehension question after each sentence that focused on the object of the relative clause verb. Again, all relative clauses had an animate subject and an animate object to control for potential animacy effects (Traxler et al., 2005). Forty-two fillers were added (including other subordinates like subject relative clauses, adverbial clauses, complement clauses and coordinate clauses).

Each pair of verbs was used for two different items, allowing participants to see both the subject-biased verb and the object-biased verb in different items. Moreover, to avoid semantic biases beyond the verbs, we created two versions of each item. In the second version, we exchanged the referents occupying the role of subject and object so that the object of the relative clause (*the professor that the assistant contacts*) became the subject in the other version (*the assistant that the professor contacts*), leading to two counterbalanced versions of the experiment. For the statistical analysis, we considered both versions of each item as one unique item.

#### Procedure.

The procedure was the same as in the previous acceptability judgment experiments.

### Results

#### Methods of Analysis.

Dependent variables were the ratings for acceptability judgments (from 1 to 11) and answers to comprehension questions (0 for incorrect answers and 1 for correct answers). The independent variables were coded as follows: 1 for subject-biased verb and 0 for object-biased verb for Verb Type, and 1 for agent-patient verbs and 0 for stimulus-experiencer verbs for Thematic Role, as well as 1 for subject relative clause and 0 for object relative clause for Clause Type. We applied mean-centered coding by scaling so that 0 coded condition approached −0.5 and 1 coded condition approached 0.5. In accordance with the maximal model justified by the experimental design (Barr et al., 2013), for the participants variable, we included random slopes for verb bias, clause type and thematic role as well as their interactions, and for the items variable, we included random slopes for verb bias and clause type.

#### Hypothesis.

If implicit causality plays a role in subject and object relative clause difficulty beyond thematic roles, object relative clauses with subject-biased verbs should be considered less acceptable and be less accurately understood than object relative clauses with object-biased verbs, independent of the thematic roles associated with the verb. As in previous experiments, we expect causality biases to play a minor role for subject relative clauses.

#### Acceptability Judgments.

**Figure 8.**
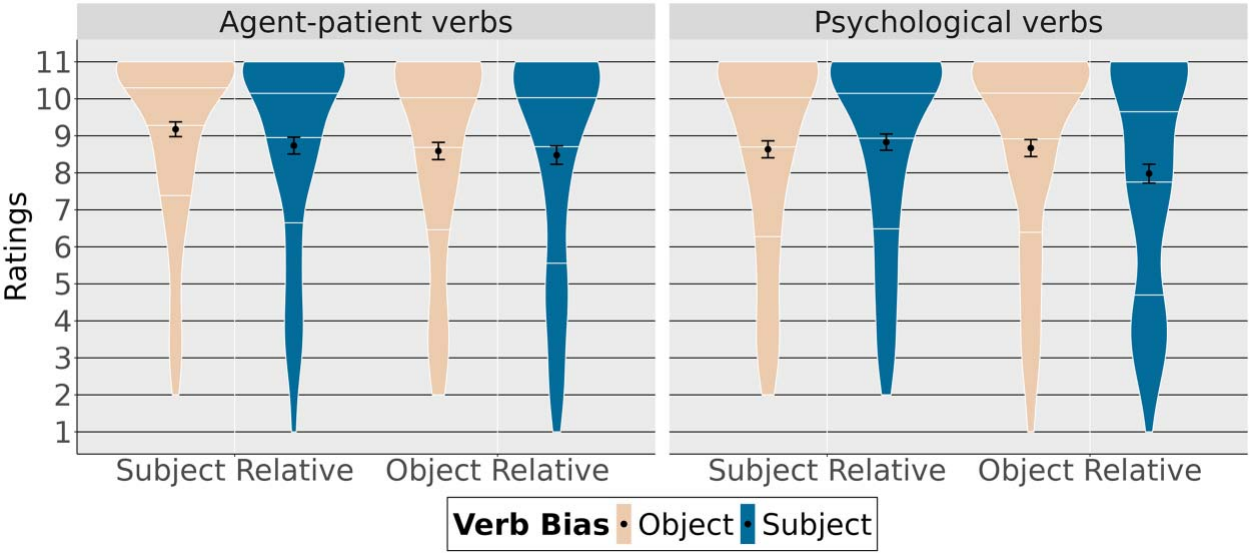
Ratings depending on thematic role and verb bias.

Violin plots in Figure 8 show the mean acceptability judgments depending on the relative clause and verb bias (subject/object) for agent-patient verbs and psychological verbs.

Table 14 summarizes the fixed effects. Ratings appeared to be lower with subject-biased verbs than with object-biased verbs (moderate probability of an effect of verb bias: P(*β* < 0) = 0.94). Subject relative clauses were more acceptable than object relative clauses (high probability of an effect of clause type, P(*β* > 0) = 1). The results also suggest that relative clauses with agent-patient verbs might be more acceptable than relative clauses with psychological verbs (moderate probability of an effect, P(*β* > 0) = 0.82). Finally, sentences with subject-biased verbs were rated lower than sentences with object-biased verbs but this was especially true for agent-patient verbs in subject relative clauses and for psychological verbs in object relative clauses (high probability of an interaction between the three factors, P(*β* < 0) = 0.98).

**Table 14.** Results from the ordinal model (acceptability judgment)

	Effect	$\hat{\beta }$	**P**(*β* > 0)	**P**(*β* < 0)	**CrI**
*Main Analysis*	Verb Bias	−0.32		0.94	[−0.73, 0.10]
	Clause Type	0.57	1		[0.28, 0.86]
	Role	0.24	0.82		[−0.28, 0.74]
	Verb Bias:Clause Type	0.22	0.79	0.21	[−0.34, 0.78]
	Verb Bias:Role	−0.11	0.39	0.61	[−0.89, 0.66]
	Role:Clause Type	−0.15	0.33	0.67	[−0.85, 0.51]
	Verb Bias:Role:Clause Type	−1.26		0.98	[−2.50, −0.03]
*Agent-patient verbs and subject relatives*	Verb Bias	−0.56		0.94	[−1.28, 0.17]
*Agent-patient verbs and object relatives*	Verb Bias	−0.11	0.35	0.65	[−0.74, 0.53]
*Psychological verbs and subject relatives*	Verb Bias	0.16	0.69	0.31	[−0.49, 0.83]
*Psychological verbs and object relatives*	Verb Bias	−0.60		0.95	[−1.36, 0.15]

#### Comprehension Questions.

**Figure 9.**
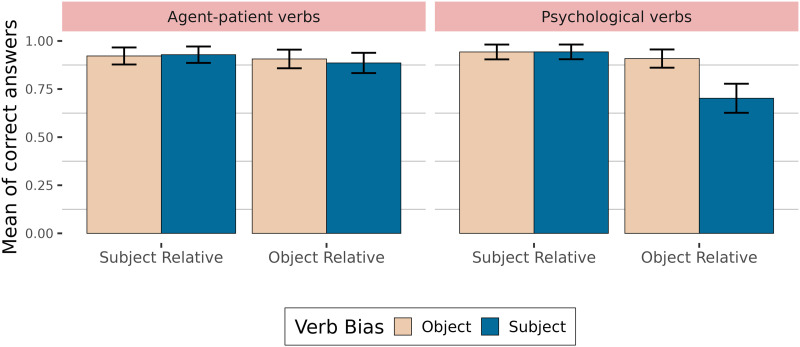
Accuracy depending on thematic role and verb bias.

Proportions of correct answers to comprehension questions are shown in Figure 9.

Table 15 summarizes the fixed effects. We found that accuracy was lower for relative clauses with subject-biased verbs than with object-biased verbs (high probability of an effect of verb bias, P(*β* < 0) = 0.98). Additionally, subject relative clauses were better understood than object relative clauses (high probability of an effect, P(*β* > 0) = 0.95). The results suggest that verb bias do not play the same role depending on the clause type and the thematic role (moderate probability of interaction, P(*β* < 0) = 0.89), indicating that accuracy appeared to be lower for subject-biased verbs in object relative clauses for agent-patient verbs, but specifically for psychological verbs.

**Table 15.** Results from the binomial model (accuracy)

	Effect	$\hat{\beta }$	**P**(*β* > 0)	**P**(*β* < 0)	**CrI**
*Main Analysis*	Verb Bias	−0.62		0.98	[−1.28, −0.004]
	Clause Type	0.80	0.95		[−0.20, 1.82]
	Role	0.28	0.75	0.25	[−0.54, 1.14]
	Verb Bias:Clause Type	1.40	0.98		[0.09, 2.76]
	Verb Bias:Role	0.89	0.92		[−0.38, 2.15]
	Role:Clause Type	−1.21		0.93	[−2.86, 0.43]
	Verb Bias:Role:Clause Type	−1.54		0.89	[−4.13, 1.04]
*Agent-patient verbs and subject relatives*	Verb Bias	0.03	0.52	0.48	[−1.66,1.63]
*Agent-patient verbs and object relatives*	Verb Bias	−0.90		0.85	[−3.03, 0.68]
*Psychological verbs and subject relatives*	Verb Bias	−0.05	0.49	0.51	[−2.09, 1.90]
*Psychological verbs and object relatives*	Verb Bias	−2.20		1	[−3.69, −0.98]

### Discussion

In this experiment we replicated an overall effect of implicit causality on object relative clauses accuracy but not on acceptability where the expected effect was only found for stimulus-experiencer and experiencer-stimulus verbs (i.e. psychological verbs). This suggests that thematic roles may interact on a certain level with implicit causality. As mentioned earlier, when we selected subject and object-biased verbs from the corpus according to the selected thematic roles, we had to compromise on the difference between subject bias and object bias, especially for agent-patient verbs (on average 31 and −77 for subject-and object-biased agent-patient verbs, and 75 and −65 for stimulus-experiencer and experiencer-stimulus verbs). We noticed that agent-patient verbs were mostly rather object-biased than subject-biased, and the difference between subject bias and object bias was smaller than we would have liked. We also found an effect of verb bias for agent-patient verbs only in subject relative clauses in an unexpected direction for acceptability judgments only: relative clauses with subject-biased verbs were less acceptable than relative clauses with object-biased verbs. We leave this surprising effect for future research.

As discussed before, thematic roles are often defined including aspects of causality. Gennari and MacDonald (2008) and Gennari and MacDonald (2009) found that stimulus-experiencer verbs make object relative clauses particularly hard to process. The stimulus-experiencer verbs used in Gennari and MacDonald (2009) (and mostly coded as cause-experiencer verbs in Gennari & MacDonald, 2008) typically have a subject that can be assumed to have caused the event by some action or property. As mentioned earlier, many of the verbs used are so-called psychological verbs with what Bott and Solstad (2014), among many others, call stimulus-experiencer verbs. Gennari and MacDonald (2009) compare these stimulus-experiencer verbs to agent-patient verbs. In our experiments, we chose our verbs based on the implicit causality bias that had been independently tested in a separate series of experiments.

**Figure 10.**
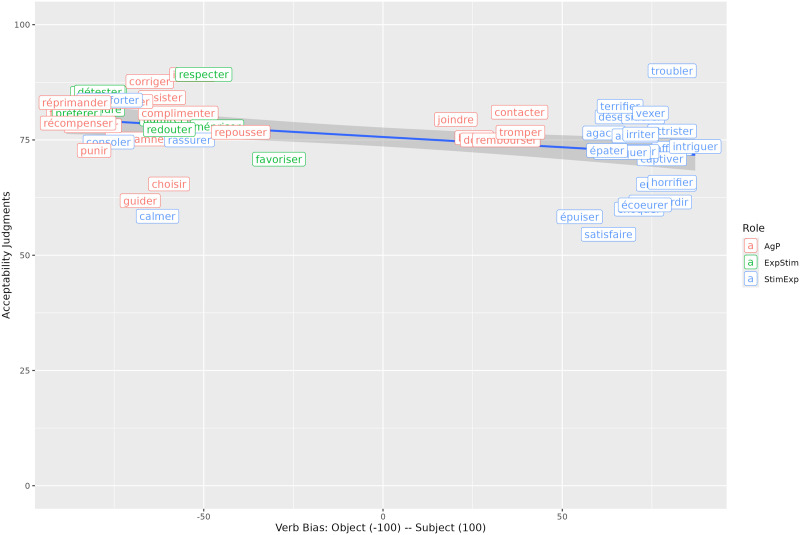
Acceptability based on bias and thematic roles.

**Figure 11.**
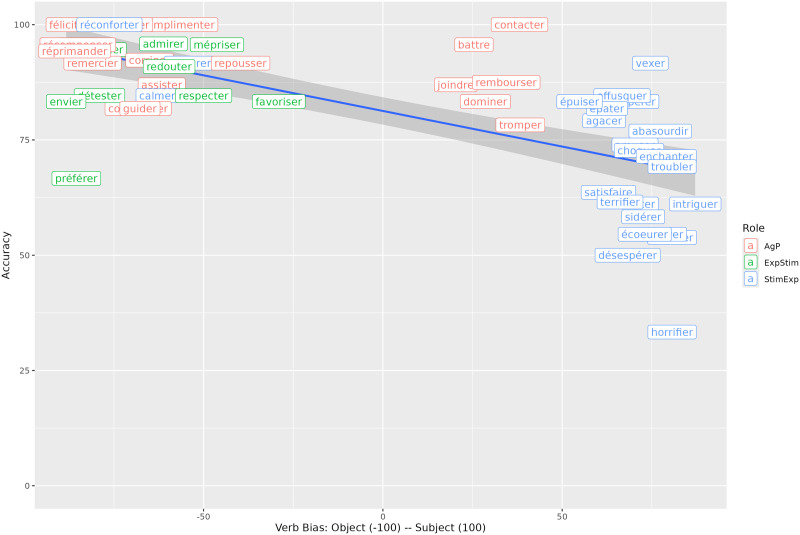
Accuracy based on bias and thematic roles.

In order to shed more light on the correlation of causality and thematic roles, we combined the data from Experiment 1 and Experiment 4. Figures 10 and 11 show the effect of bias on judgments and accuracy of object relative clauses respectively with verb classes colour coded.

Bott and Solstad (2014) argue that the stimulus argument in psychological verbs (but also the evocator argument in agent-evocator verbs) are semantically underspecified with an open slot for a specific explanation. This is, we would argue, what makes these arguments more topical and thus influences relative clause processing. For agent-patient verbs, Bott and Solstad (2014) argue that the missing explanation is based on missing information about a presupposition. When we hire somebody, we typically assume that we do this because the person has some quality or skill to be hired for. A possible explanation for the fact that object-biased agent-patient verbs do not make understanding object relative clauses easier may be that the missing information is less salient than for stimulus-experiencer verbs. Biases for agent-patient verbs may be more related to world knowledge than verb semantics as suggested by van den Hoven and Ferstl (2018).

A clear effect of bias has been established across experiments, for both, acceptability judgments and accuracy (for accuracy: $\hat{\beta }$ = −0.01, 95% CrI = [−0.02, −0.01], P(*β* < 0) = 1; for acceptability: $\hat{\beta }$ = −0.002, 95% CrI = [−0.003, 0.000], P(*β* < 0) = 0.97). However, we can see that, in line with Gennari and MacDonald (2009), stimulus-experiencer verbs are mostly subject-biased and thus contribute most strongly to lower acceptability and accuracy. On the other hand, object-biased verbs are not just agent-patient verbs but can be experiencer-stimulus verbs and even some stimulus-experiencer verbs. These verbs come on average with higher acceptability and accuracy of object relatives. This indicates that implicit causality and thematic roles are linked at a certain level. In line with Bott and Solstad (2014), we suggest that at least part of the underlying reason for thematic role effects is the expectancy of an as yet underspecified explanation for an argument that makes this argument more topical. How strongly an explanation is missed may correlate with thematic roles, leading to differences across verb classes.

## GENERAL DISCUSSION

In this paper, we revisited the debate regarding the asymmetry between subject and object relative clauses focusing on the influence of the implicit causality of the relative clause verb.

In Experiments 1 and 2, we manipulated the implicit causality of the relative clause verbs in an acceptability judgment task and a self-paced reading task. Results from acceptability judgments and comprehension questions confirmed our predictions: object relative clauses containing subject-biased verbs were the most difficult to accept and to understand. Surprisingly, results from the self-paced reading task (Experiment 2) showed a different pattern for reading times: relative clauses with subject-biased verbs led to an increase in reading times, regardless of the construction. Still, we found the same patterns as in Experiment 1 for comprehension questions and answering times for questions. Regarding the online processing of implicit causality, it may be the case that online reading times and final acceptability and comprehension are not fully affected by the same factors. Our results suggest that the integration of implicit causality in the sentence does not occur at early processing stages, but rather in post-interpretative processing (Caplan & Waters, 1999), namely in acceptability and comprehension questions. This is quite different from other factors, such as animacy which appears to affect early processing, i.e., the interpretative processing. This can be explained by the fact that the integration of implicit causality in the sentence implies an effect on the verb as well as its two arguments, while animacy is marked on the noun itself. It could also be the case that the implicit causality of the verb is interpreted as a result of good-enough processing (Ferreira et al., 2002), and it is only integrated in the semantic representation of the sentence when participants are asked to provide their interpretation. As suggested by one of our reviewers, this may partly be a memory-based effect in that the representation of the sentence that has to be accessed for acceptablity judgments and comprehension questions may be more stable when causality biases are aligned with aboutness topics. We leave this question of differences between early and late processing for future research.

As for Experiments 3 and 4, we tested other factors that may be at play and explain our previous results, as suggested by syntax-based, memory-based or semantic/discourse-based theories. Experiment 3 (acceptability judgements) tested a syntactic factor that could have explained the results, namely the intervening subject between the filler and the gap in the relative clause. We manipulated the subject position in object relative clauses in French, and we found that subject-biased verbs were still the most difficult to understand, independent of the position of the subject. These results could not only be attributed to intervention or linear order effects, meaning that implicit causality is not just a secondary factor. In Experiment 4, we manipulated thematic roles and implicit causality, again using an acceptability judgment task. If thematic roles were the main factor in relative clause acceptability, relative clauses with agent-patient verbs should have been more acceptable and understood than relative clauses with stimulus-experiencer verbs overall. This is, however, not what we found. We did find an implicit causality effect in the expected direction only for stimulus-experiencer verbs in object relative clauses though, suggesting that, while implicit causality does play a role beyond thematic roles, implicit causality and thematic roles appear to be correlated.

It is important to note that subject-biased verbs appear to cause difficulties in reading times and acceptability across all experiments even for subject relative clauses (though much less so than for object relative clauses). Regarding our hypothesis, we did not expect a strong interference of implicit causality for subject relative clauses because of the special status of the subject as the default aboutness topic. We suggest that type frequency might be at play for the integration of implicit causality in the sentence. Subject-biased verbs are generally less frequent than object-biased verbs (Gennari & MacDonald, 2009). However, more research on this question will be necessary.

Overall, implicit causality including its reflection in thematic roles is a factor to take into account that directly interacts with the relative clause function. A still open question is why this is the case. A first hypothesis that may suggest an explanation is the topichood hypothesis suggested by Mak et al. (2006) and Roland et al. (2012). Mak et al. (2008) and Roland et al. (2012) provide evidence that the discourse topicworthiness of the internal subject can make object relative clauses easier to process. However, as mentioned in the introduction, it remains unclear why increased topicworthiness of the internal subject does not increase competition with the antecedent. This would be the prediction that any interference-based theory would make. The advantage of an object relative clause with a discourse topic as the subject cannot be explained solely by its topicworthiness unless a clear distinction is made between topicworthiness as the aboutness topic of the relative clause and topicworthiness as the discourse topic.

Memory-based, especially competition-based approaches such as Gordon et al. (2006) or Lewis et al. (2006) may contribute to an explanation. Indeed, a competition could arise between the antecedent and the relative internal noun phrase, modulated by the interference of the implicit causality of the relative clause verb. However, these theories do not provide an explanation for why implicit causality should have this effect. Moreover, these accounts depend on the linear intervention of the internal subject, but the results from Experiment 3 show that the competition between syntactic function is independent of the position of the subject in the relative clause.

We suggest another explanation for the interference of the implicit causality of the verb based on the function of a relative clause, the Aboutness hypothesis: a relative clause is more acceptable and easier to understand when everything contributes to making the head its optimal aboutness topic.

The difficulty to understand a relative clause may depend on the relationship between the antecedent and the information provided by the relative clause, i.e., how well the antecedent serves as the optimal candidate for what the relative clause is about, or its “aboutness topicworthiness,” to use terminology from Mak et al. (2006). Comprehension will be more difficult when there is a conflict between the antecedent as the default aboutness topic and what the relative clause suggests to be about. The implicit causality of the verb can interfere in this relation. Verb bias can either reduce or increase the conflict of topicworthiness between the antecedent and the relative clause internal noun phrase by making the internal noun phrase more or less salient. In particular, object relative clauses with subject-biased verbs will increase comprehension difficulty because of conflicting information with respect to what the relative clause is “about” contrary to object relative clauses with object-biased verbs.

Different from Mak et al. (2006), we do not focus our hypothesis on the discourse topic, but rather on the function of the relative clause that modifies the antecedent. Our explanation is still compatible with their results: Discourse topics are given, and thus less likely to be aboutness topics of restrictive relative clauses. They would therefore compete with the status of the antecedent as the aboutness topic to a lesser degree.

By making a clear distinction between discourse topics and aboutness topics and focusing on the function of the relative clause, we suggest the Aboutness hypothesis as a tentative explanation of the way implicit causality interferes in the acceptability and comprehension of relative clauses. More experiments are surely necessary to test this hypothesis in more detail.

## CONCLUSION

To conclude, we investigated the role of implicit causality in subject and object relative clause processing in French. Experiment 1 showed that object relative clauses with subject-biased verbs were the least acceptable and understood. Experiment 2 showed an effect of subject-biased verbs but no interaction with the function of the relative clause, meaning that this factor occurs at a later level of processing. Acceptability judgments from Experiments 3 and 4, manipulating the subject position in object relative clauses and thematic roles suggested that syntactic and thematic role factors alone cannot explain object relative clause processing. Alongside other accounts, we suggest the Aboutness hypothesis, assuming that factors more in line with information structure linked to the function of relative clauses and to implicit causality need to be taken into account to understand the asymmetry in subject and object relative clause processing.

## ACKNOWLEDGMENTS

We also would like to thank Jean-Marie Marandin and Lisa Brunetti for very helpful discussions.

## FUNDING INFORMATION

This work was partially supported by the AAP Jeunes chercheur·se·s-Néo MCFs 2021 from Paris 8 University and Structures Formelles du Langage, as well as by a public grant overseen by the French National Research Agency (ANR) as part of the program “Investissements d’Avenir” (reference: ANR-10-LABX-0083). It contributes to the IdEx Université de Paris – ANR-18-IDEX-0001.

## DATA AVAILABILITY STATEMENT

Experiment materials, as well as data analysis files, are available via https://osf.io/53dzc/.

## ETHICS STATEMENT

The study was approved by the Comité d’Ethique pour les Recherches en Santé at the University Paris Sorbonne Cité (CERES; Application Number: 2018-34).

## Notes

^1^ The antecedent is also referred to as the head in other linguistic theories. Since there is some debate over what the head of a relative clause is, we will use the term “antecedent”.^2^ Other positions in the relative clause can be relativized depending on the language (Keenan & Comrie, 1977).^3^ Trees in this paper are drawn according to minimality syntactic tradition when referring to the Relativized Minimality hypothesis.^4^ This theory does not predict a subject advantage for all languages, see for example, Hsiao and Gibson (2003). These results are quite controversial, though (Jäger et al., 2015; Vasishth et al., 2013).^5^ We will keep the terms stimulus-experiencer and agent-patient in the rest of the paper to be more in line with the linguistic literature.^6^ Gibson (2001) makes similar prediction assuming that discourse-old referents are easier to process.^7^ Acceptability judgments on a scale of 1 to 10 are commonly used in France, particularly for surveys and school exams. Studies conducted in French often employ this scale, as it is more familiar to French participants.^8^ Object relative clauses with postverbal subjects cannot be confounded with subject relative clauses because of the difference in the relativizer (*qui* for subject relative clauses and *que* for object relative clauses).^9^ It could even be argued that French object relative clauses with postverbal subjects should generally be more acceptable than with preverbal subjects. However, two factors play against this assumption. First, the usage of object relative clauses with postverbal subject is more constrained. While they can be as acceptable as object relative clauses with preverbal subjects in the right context (Pozniak et al., 2021), postverbal subjects are generally less frequent. For object relative clauses, this is particularly true for subjects with agentive properties as in our experiments. This may affect their general acceptability. Second, the only difference between a subject relative clause and an object relative clause with a postverbal subject is the distinction between *que* for object relative clauses vs. *qui* for subject relative clauses. Following the logic of noisy channel models (Gibson et al., 2013; Jurafsky & Martin, 2008), object relative clauses with postverbal subjects may be misunderstood as subject relative clauses thus leading to generally lower comprehension accuracy. Neither of these two factors is, however, expected to interact with implicit causality biases.^10^ As for agent/patient verbs, we could not find all the verbs in the corpus from Ferstl et al. (2011), even though we could notice that the verb bias was less on the subject than on the object (a mean of 6 for 10 verbs).^11^ Our Experiment 2, shows, however, that the processing disadvantage may not really be related to object relative clause processing since we found a similar pattern for subject relative clauses.^12^ Bott and Solstad (2014) call verbs like *criticize* agent-evocator verbs.
